# Structural Basis for Species Selectivity in the HIV-1 gp120-CD4 Interaction: Restoring Affinity to gp120 in Murine CD4 Mimetic Peptides

**DOI:** 10.1155/2011/736593

**Published:** 2012-01-19

**Authors:** Kristin Kassler, Julia Meier, Jutta Eichler, Heinrich Sticht

**Affiliations:** ^1^Institute of Biochemistry, Friedrich-Alexander-Universität Erlangen-Nürnberg, Fahrstraße 17, 91054 Erlangen, Germany; ^2^Department of Chemistry and Pharmacy, Friedrich-Alexander-Universität Erlangen-Nürnberg, Schuhstraße 19, 91052 Erlangen, Germany

## Abstract

The first step of HIV-1 infection involves interaction between the viral glycoprotein gp120 and the human cellular receptor CD4. Inhibition of the gp120-CD4 interaction represents an attractive strategy to block HIV-1 infection. In an attempt to explore the known lack of affinity of murine CD4 to gp120, we have investigated peptides presenting the putative gp120-binding site of murine CD4 (mCD4). Molecular modeling indicates that mCD4 protein cannot bind gp120 due to steric clashes, while the larger conformational flexibility of mCD4 peptides allows an interaction. This finding is confirmed by experimental binding assays, which also evidenced specificity of the peptide-gp120 interaction. Molecular dynamics simulations indicate that the mCD4-peptide stably interacts with gp120 via an intermolecular **β**-sheet, while an important salt-bridge formed by a C-terminal lysine is lost. Fixation of the C-terminus by introducing a disulfide bridge between the N- and C-termini of the peptide significantly enhanced the affinity to gp120.

## 1. Introduction

AIDS is one of the most dramatic infection diseases in humans, not only in developing countries but also in the western world. The disease is caused by HIV-1, which infects CD4+ cells, that is, T helper cells and macrophages. The first step in cell invasion is binding of the HIV-1 envelope protein gp120 to the cellular surface receptor CD4 [1]. Attachment inhibitors are a class of anti-HIV-1 drugs that interfere with this very first step of viral entry and include derivatives of natural ligands, antibodies, and small synthetic compounds [2].

**Figure 1:**
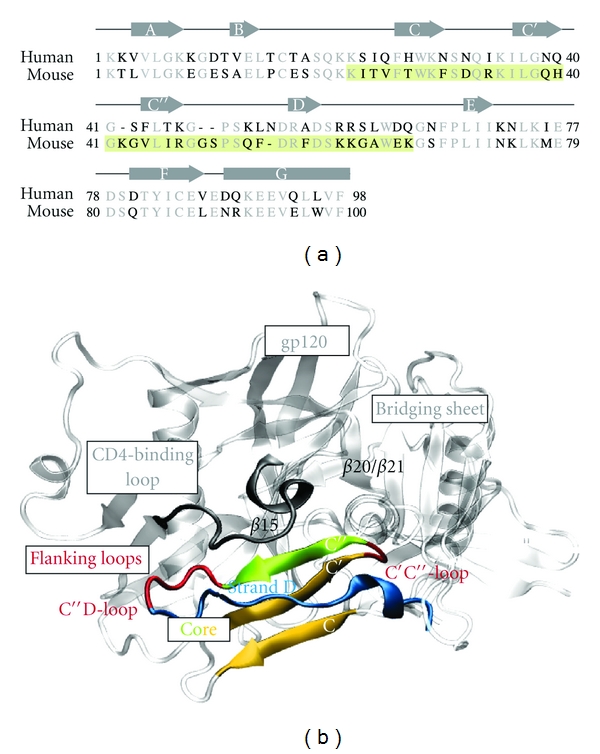
Sequence comparison and interactions of CD4. (a) Sequence alignment of human and murine CD4 in the region of the immunglobulin-like domain 1 (D1). Nonconserved and conserved residues are shown in black and gray, respectively. The stretch covered by the mCD4-M (residues 22 to 66) peptide is highlighted in green. The position of *β*-strands is indicated as arrows above the sequence. This alignment represents the final sequence alignment used for homology modeling. Information about alternative alignments and the quality of the resulting models is given in Supplementary Material available online at doi:10.1155/2012/736593. (b) Region of interaction between CD4 and gp120. gp120 is shown in light gray; the different structural parts of CD4 are color coded as follows. The triple-stranded *β*-sheet of the CD4 domain D1 (termed “core”) is shown in yellow and green. The green color highlights strand C*″* that forms an intermolecular *β*-sheet with strand *β*15 of gp120. Loops connecting the *β*-strands of the core are shown in red, and the C-terminally adjacent strand D is colored in blue.

The structural details of the gp120-CD4 interaction are known from several complex crystal structures [3–7], thus providing the basis for a rational design of inhibitors. The T cell receptor CD4 belongs to the immunoglobulin superfamily and consists of a short cytoplasmatic tail, a single transmembrane helix, and four tandem immunoglobulin repeats (D1–D4) [8]. Residues 25–64 of domain D1 constitute the binding site for gp120 [4] (Figure 1(a)). The gp120-binding site is composed of a triple-stranded antiparallel *β*-sheet (strands C, C′, and C*″*) followed by strand D that exhibits a rather poor *β*-strand geometry. In total, 22 residues of CD4 contact 26 amino acids of gp120 [4]. Strand C*″* of CD4 establishes hydrogen bonds to strand *β*15 of the gp120 CD4-binding loop. This leads to the extension of the three stranded core *β*-sheet of CD4 (C-C′-C*″*) by one strand of gp120, establishing an intermolecular *β*-sheet at the interface. Key residues in human CD4 for its interaction with gp120 are F43 and R59, located in strand C*″* and D, respectively (Figure 1(b)). F43 binds into a hydrophobic cavity of the gp120 surface, and R59 forms a salt-bridge with D368 of gp120 [4]. The significance of F43 and R59 for gp120 recognition is supported by mutational data revealing that mutation of F43 [9–11] or R59 [11, 12] to alanine or glycine reduces gp120-binding. One of the most devastating mutations, F43A, leads to a 500-fold reduction of gp120-binding [11].

This detailed structural and functional knowledge has been exploited in the past to derive peptides from human CD4 (hCD4) that bind to gp120, thereby inhibiting the gp120-CD4 interaction [13–15]. In this context, CD4 mimics, in which key residues for the interaction with gp120 were grafted on the scorpion toxin scyllatoxin, represent a very potent group of compounds [13].

Apart from their biomedical significance as inhibitors of protein-protein interactions, synthetic mimics of protein binding sites are also valuable tools for the exploration of these interactions at the molecular level. The molecular basis of the fact that mice cannot be infected with HIV [16] lies in the lack of affinity of HIV gp120 to murine CD4 (mCD4) [17–19], in spite of a fairly high sequence homology (55% identity) between the first extracellular domain (D1) of murine and human CD4, which contains the binding site for gp120.

Therefore, the first goal of our work was to understand the structural origin for this lack of affinity of gp120 to mCD4. In a second step, we could show by a combination of computational predictions and binding assays that an mCD4 mimetic peptide comprising residues 22–66 can bind gp120. The mode of this peptide-gp120 interaction has also been studied in greater detail by molecular dynamics simulations, with the aim to propose, generate, and evaluate modified mCD4 mimetic peptides with improved affinity to gp120.

## 2. Material and Methods

### 2.1. Peptide Synthesis and Binding Experiments

Peptide synthesis and binding assays were done essentially as described earlier [20]. Briefly, peptides (refer to Table 2 for sequences) were synthesized as C-terminal amides by Fmoc/t-Bu-based solid-phase synthesis using an automated multiple peptide synthesizer and N-terminally acetylated. Cleaved peptides were purified by preparative HPLC. A cysteine residue was added to the linear peptides, enabling covalent attachment to SH binding plates in the binding assay. The C23–C65 disulfide bridge in mCD4-M** was formed by air oxidation. This peptide, as well as a second copy of hCD4-M, was equipped with a His6-tag, enabling attachment of the peptides to Ni-NTA assay plates. Binding assays were performed in SH-binding or Ni-NTA microtiter plates, respectively, to which the peptides were coated at 1 *μ*M. Binding of gp120 (0.5 *μ*g/mL) to the peptides was detected using a primary anti-gp120 antibody, followed by a peroxidase-labeled secondary antibody in conjunction with a chromogenic substrate, enabling a quantitative colorimetric readout. Relative affinities were calculated by dividing the absorbances obtained for each peptide by the absorbance obtained for hCD4-M, which enabled comparison of results from different assays using different chemistries for peptide attachment to the plates.

### 2.2. Homology Modeling of Murine CD4

A model of mCD4 consisting of domains D1 and D2 was generated based on the crystal structure of human D1/D2–CD4 that was determined in complex with gp120 (1RZJ [3]). The murine and human D1/D2–CD4 proteins exhibit a sequence identity of 52% thus allowing the application of homology modeling techniques. Since two slightly different alignments of human and murine CD4 have been reported in the literature (Figure S1), two different models of mCD4 were constructed. For each model, the sequence of mCD4 was aligned to hCD4 protein using the SwissPDB viewer v3.7 [21] and the mCD4 structure was generated with SwissModel [22–24]. Model quality was assessed based on the QMEAN score [25, 26] provided by Swiss-Model and on further verification with PSQS [27, 28] and ProSA [29, 30]. Fitting of the mCD4 structure onto the hCD4 coordinates in the hCD4-gp120 complex yielded a model of a putative mCD4-gp120 complex. This complex was analyzed for clashes using WHAT_CHECK [31].

### 2.3. Molecular Dynamics Simulations of the gp120-mCD4(22–66) Complex

The gp120-binding properties of a peptide corresponding to residues 22–66 of mCD4 were investigated by a 100-ns molecular dynamics (MD) simulation. This simulation was based on the modeled mCD4-gp120 complex structure from which all mCD4 residues outside the 22–66 stretch were deleted. N- and C-termini of the mCD4 (22–66) peptide were blocked with acetyl and N-methyl amide groups, respectively. The protonation state of histidine residues was checked visually and if necessary either N*δ* or N*ε* protonation was chosen to ensure optimal hydrogen bonding. MD simulation was performed by the AMBER10 [32] and AMBER11 [33] suite of programs together with the force field ff99SB [34] including updated torsion potentials. Using the AMBER11 tool tleap [33], the system was neutralized with Cl^−^ ions and placed in a TIP3P [35] water box with at least 12 Å space to the box boundaries. Subsequently, the system was minimized, heated, and pressure equilibrated according to a previous simulation protocol [36]. Minimization was performed in three steps. Initially, only solute molecules were minimized while restraining protein atoms with a force constant of 500 kcal mol^−1^ Å^−2^. Next, side chains were relaxed while forcing the backbone to its initial position by applying the same force constant. Finally, all constraints were removed allowing the whole system to relax. Each step involved 250 steps of steepest descent followed by 250 steps of conjugate gradient minimization. The minimized structure was subjected to MD simulation at 310 K using a time step of 2 fs and periodic boundary conditions. SHAKE [37] was applied to fix all hydrogen involving bonds. After heating the system from 10 to 310 K (100 ps, NVT ensemble), and equilibrating it at constant pressure (400 ps, NPT ensemble at 1 bar), it was subjected to a 100-ns production phase carried out with Amber10 [32]. The obtained trajectory was processed utilizing the AMBER11 tool ptraj. Structure visualization was performed with VMD [38].

## 3. Results and Discussion

### 3.1. Structural Basis for the Lack of gp120-Binding by Murine CD4

Previous experiments have revealed that the mCD4 protein does not interact with HIV-1 gp120 [16–18]. To identify the structural properties responsible for this lack of interaction, a homology model of the mCD4-gp120 complex was generated.

Homology modeling of mCD4 is hampered by the fact that slightly different sequence alignments between hCD4 and mCD4 have been reported in the past [39–41], which differ in the alignment of the C*″* D-loop (Figure S1). To identify the alignment, which is more suitable for comparative modeling, two structures were generated based on the different sequence alignments and compared with respect to their model quality (Table S1, Figures S2, and S3). Interestingly, both models exhibit clashes at the mCD4-gp120 interface, which can, therefore, be considered as an intrinsic feature impeding interaction of the complex regardless of the CD4 sequence alignment used. Therefore, all further analysis is based on the murine model 1, which exhibits the more favorable structural properties (Table S1).

**Figure 2:**
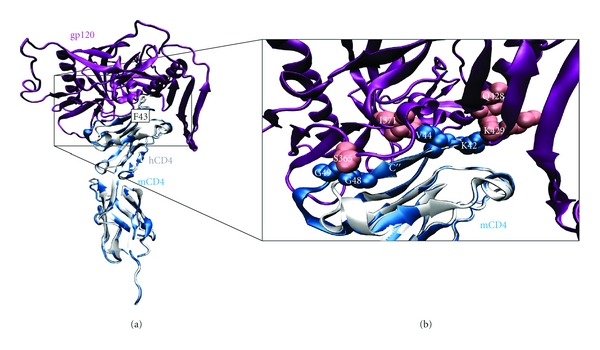
Clashes of murine CD4 with gp120. (a) Overlay of the unbound mCD4 model onto the hCD4-gp120 complex structure 1RZJ [3]. (b) Close-up of the clash area. Clashes are predominantly found for residues in the loops flanking *β*-strand C*″* (G48, G49, K42) and for V44. The respective loops are longer in mouse compared to human CD4 (cf. alignment Figure 1(a)). Strand C*″* is labeled. hCD4, mCD4, and gp120 are colored white, blue, and pink, respectively. Clashing residues of mCD4 and gp120 are shown in blue and pink spacefill.

The overlay between the murine and human CD4-gp120 complexes reveals the high overall structural similarity (Figure 2(a)), while a detailed inspection of the interface highlights several clashes present in the mCD4-gp120 complex (Figure 2(b)). Clashing residues are predominantly located in the loops flanking *β*-strand C*″* (C′C*″*-loop, C*″*-D-loop), two regions that show sequence insertions in mouse compared to human CD4 (Figure 1). Structural analysis identifies several residues that form clashes >0.8 Å (Table 1). The largest intermolecular clash is found between G49, located in the C*″*D-loop, and S365, located in the CD4-binding loop of gp120, with a size of 1.95 Å. An attempt to remove this clash by a 500-ps MD simulation in explicit solvent resulted in a very unfavorable backbone geometry of G49: *φ* = +159.0° and *ψ* = −86.6° (compared to *φ* = −81.2°, *ψ* = +27.2° in the initial model). Furthermore, K42 and V44 contribute significant clashes to the interface. K42, an insertion in the C′C*″*-loop, clashes with two residues of the bridging sheet of gp120. The larger of the clashes is formed between the K42 (mCD4) and K429 (gp120) side chains (Table 1, Figure 2) indicating that electrostatic repulsion additionally counteracts the mCD4-gp120 complex formation.

Valine44, the sequential equivalent to F43 in hCD4, overlaps with I371. Interestingly, the described clashes affect residues and positions where human and mouse sequences diverge. Those findings are in line with previous studies that demonstrated a complete loss of binding affinity following K- and GS-insertion in C*″*-flanking loops of hCD4 [39]. In conclusion, clashes of the loops flanking strand C*″*, that are longer in mCD4, are most likely the major reason for impeded gp120-binding of mCD4.

The fact that gp120 mainly forms clashes with the longer loops of mCD4 prompted us to investigate whether these clashes might be removed in an mCD4 mimetic peptide (mCD4-M) due to its larger conformational freedom. Interestingly, energy minimization is sufficient to remove all intermolecular clashes (>0.4 Å) between such a peptide and gp120. In order to ensure that this finding is not an artifact of the applied force field, the respective peptide was synthesized and its gp120-binding affinity was verified experimentally.

### 3.2. Experimental Investigation of the gp120-Binding of CD4-Derived Peptides

**Figure 3:**
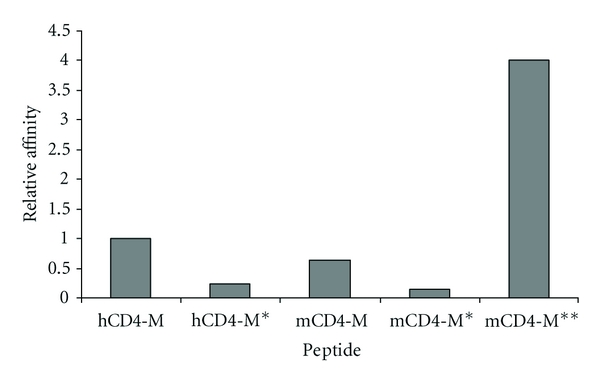
Binding affinities of CD4 peptides. Relative affinities (hCD4-M = 1) to HIV-1 gp120 of peptides mimicking the putative gp120-binding site of human (hCD4-M) and murine CD4 (mCD4-M), as well as peptide variants in which the hot spot residues were replaced by alanine (hCD4-M* and mCD4-M*, resp.), and a cyclized mCD4 mimetic peptide (mCD4-M**, see Table 2 for peptide sequences).

Peptides comprising residues 22–66 of mCD4 (termed mCD4-M) and 22–64 of hCD4 (termed hCD4-M), respectively, were synthesized and their gp120-binding affinities experimentally determined (Table 2; Figure 3). This experiment shows that mCD4-M is indeed capable of binding to gp120, and that this interaction is only slightly weaker than that of the homologous hCD4-M peptide.

 To verify that the binding of the peptides is specific, variants (hCD4-M*, mCD4-M*; Table 2) were generated that address the hot spot residues crucial for gp120-binding known from the gp120-CD4 crystal structures [3–7]. In hCD4-M, F43 and R59 were replaced by alanine. Due to sequence diversity, the corresponding residues in mCD4-M are V44 and K61 (Figure 1(a)). In these peptides, also R58 (K60 in the murine peptide) was replaced by alanine to avoid functional compensation of R59 (K61 in the murine peptide) by the sequentially adjacent basic residue, which might occur in peptides due to a higher flexibility compared to proteins. Binding of either triple variant to gp120 was notably reduced, indicating that mCD4-M specifically targets the CD4-binding site of gp120 (Figure 3). This suggests that the lack of interaction detected for mCD4 protein is not due to the sequence of mCD4 itself, but most likely to the rigid conformation imposed by the immunoglobulin fold. Therefore, the affinity can at least partially be restored in the more flexible mCD4 peptides.

### 3.3. Structural Analysis of the mCD4-M-gp120 Interaction

To understand the binding of mCD4-M in greater detail, a 100-ns molecular dynamics simulation was performed for the peptide-gp120 complex. Subsequently, the different key interaction regions were analyzed in greater detail.

**Figure 4:**
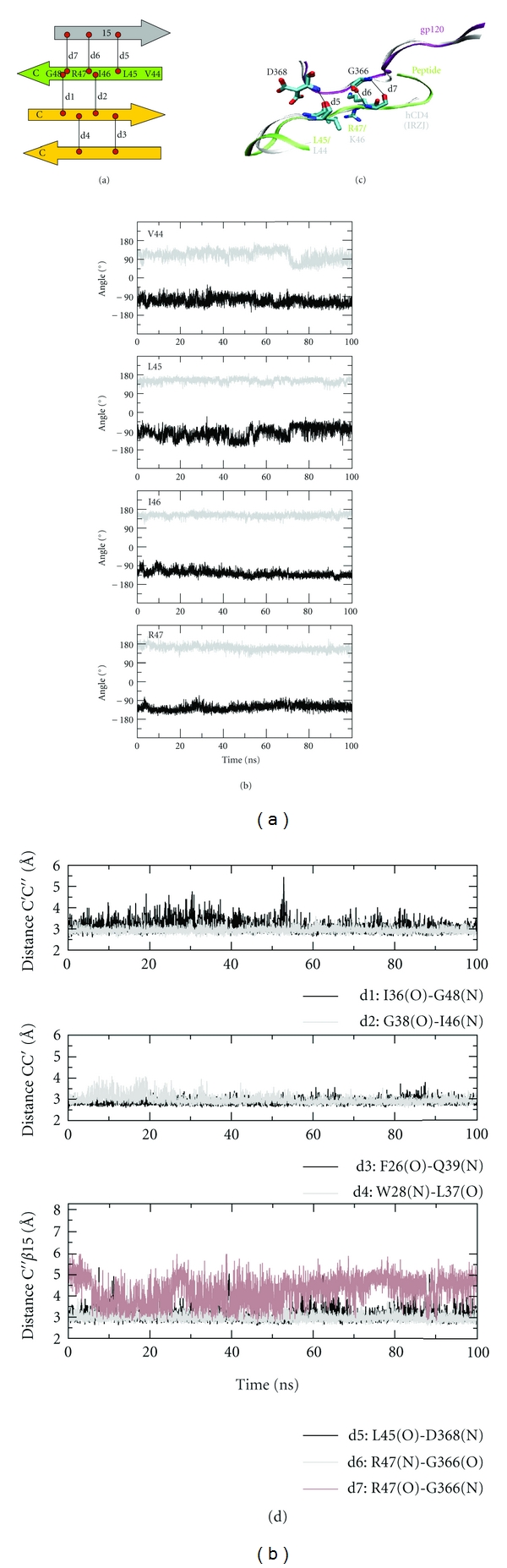
Intrinsic stability and gp120-interaction of the mCD4 peptide core. (a) Scheme of intra- and intermolecular hydrogen bonds and analyzed distances and torsion angles. Measured distances are marked d1–d7. Residues selected for torsion analysis are highlighted. (b) Dihedral angles of residues V44 to R47 are shown as a function of time. **φ** angle (C_*i*−1_-N*_i_*-C*_i_* ^*α*^-C*_i_*) in black, **ψ** angle (N*_i_*-C*_i_* ^*α*^-C*_i_*-N*_i+1_*) in gray. Distances were measured between main chain hydrogen donor and acceptor atoms. (c) Zoom into the interface and comparison of the two interfaces mCD4-peptide-gp120 and hCD4-gp120 (1RZJ). Overlay of a representative snapshot after 90 ns onto crystal structure of the gp120-hCD4 complex. Fit on residues 362–369 of *β*15. mCD4-peptide and hCD4-protein are colored in green and gray, respectively. (d) Hydrogen bond distances between *β*-strands C/C′ and C′/C*″* and intermolecular hydrogen bond distances between C*″* and *β*15.

 The peptide core contains three *β*-strands, C, C′, and C*″*, assembled in an antiparallel fashion. Stand C*″* directly interacts with strand *β*15 of gp120 (Figure 1(b), Figure 4(a)). Hence, those interactions were analyzed over the course of simulation. For the intramolecular C-C′-C*″β*-sheet, hydrogen bond distances and torsion angles were monitored over time and are plotted in Figures 4(b) and 4(d). Distances between main chain nitrogen and oxygen atoms range between 2.5–3.0 Å and are almost preserved during simulation indicating stability of core hydrogen bonding (see d1–d4 in Figure 4(d)). Analysis of torsion angles **φ**(C_*i*-1_-N*_i_*-C*_i_* ^*α*^-C*_i_*) and **ψ** (N*_i_*-C*_i_* ^*α*^-C*_i_*-N_*i*+1_) revealed an ideal *β*-sheet backbone geometry for residues V44 to R47 of strand C*″* with mean *φ* and *ψ* values ranging at negative and positive values characteristic for a *β*-sheet. Taken together, preservation of hydrogen bond distances and *β*-sheet backbone angles over time guarantees the stability of the peptide core. This intrinsic stability also is the prerequisite for the correct positioning of the peptide at the gp120-interface. Here, the peptide forms three backbone hydrogen bonds to *β*15 of the CD4-binding loop of gp120 during simulation time (see d5–d7 in Figure 4(d)). Hydrogen bonds are established between residues L45 and R47 of CD4 and D368 and G366 of gp120. One of the contacts (R47(O)-G366(N)) exhibits larger fluctuations; however, a transient hydrogen bonding can be detected over the entire simulation time (distance d7 in Figure 4(d)). The three hydrogen bonds are arranged in an antiparallel *β*-sheet fashion, thereby extending the C-C′-C*″*-central *β*-sheet by strand *β*15 across the interface. When comparing the peptide interface to the human CD4-gp120 complex of the crystal structure, a striking similarity can be noted both with respect to the *β*-sheet structure and the hydrogen bonding pattern (Figure 4(c)). The weaker third hydrogen bond detected in the mCD4-M-gp120 interface also exhibits a nonoptimal geometry in the hCD4-gp120 interface thus supporting our results [4, 42].

**Figure 5:**
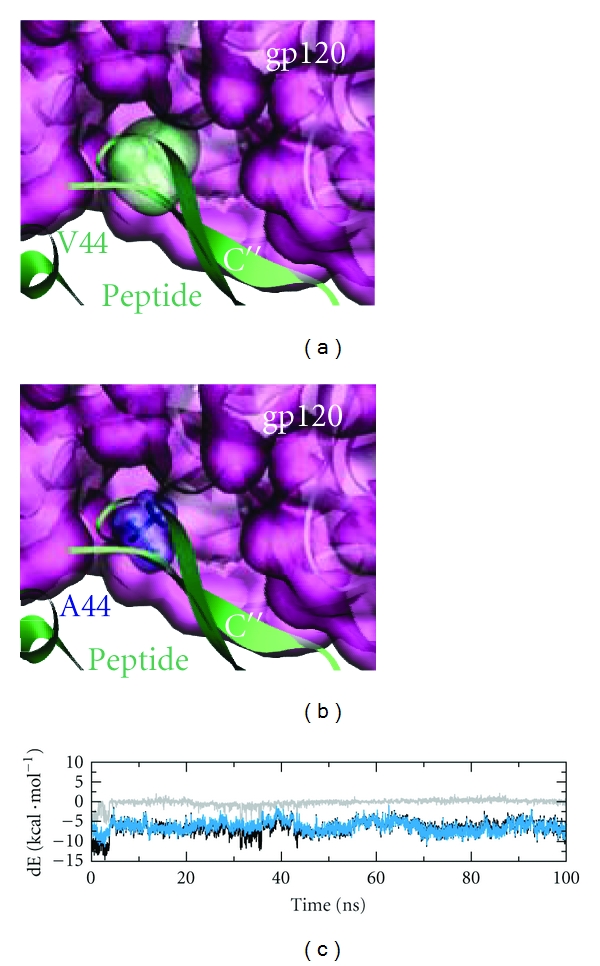
Van der Waals packing of V44 at the peptide-gp120 interface. (a) Enlargement of the interface. V44 of *β*-strand C*″* packs tightly into a pocket of the gp120 surface. (b) Replacement by alanine leads to loss of van der Waals packing. (c) Interaction energy of V44 with gp120. Total energy, electrostatic and van der Waals contribution are colored in black, gray, and blue, respectively.

 In addition to the intermolecular *β*-sheet structure, the interface is stabilized by tight van der Waals packing of V44 into a cavity of gp120 as shown in Figure 5. Tight packing is also reflected in the good interaction energy of V44 which is dominated by a strong van der Waals term (Figure 5(c)). In contrast to the mCD4 protein, where V44 causes interface clashes (Figure 2 and Table 1), the conformational plasticity of the peptide allows the residue to penetrate into the cavity. This interaction is structurally reminiscent to that of F43 in human CD4, which extends deeply into the F43-cavity of gp120 [4], forming a key contact of the hCD4-gp120 interface. An F43A mutation in human CD4 leads to a dramatic reduction (500-fold) of gp120-binding [11].

 This data is also in agreement with our experimental data for the peptides showing that replacement of F43, R58, and R59 (hCD4-M*), as well as V44, K60, and K61 (mCD4-M*) with alanine clearly diminished binding to gp120 (Figure 3). The methyl side chain of alanine cannot compensate for the missing van der Waals packing of the longer hydrophobic valine side chain, resulting in a hole in the pocket (Figure 5(b)). In conclusion, the stability of the peptide core allows the formation of an intermolecular *β*-sheet structure and a tight van der Waals packing at the gp120 interface.

**Figure 6:**
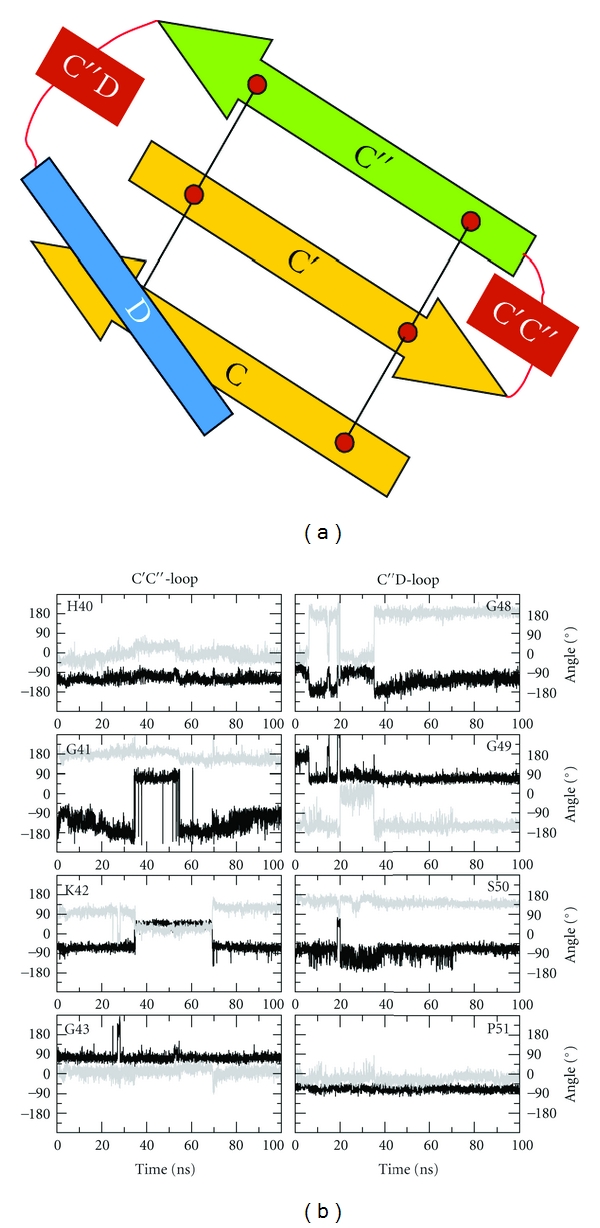
Fluctuations of torsion angles in loops flanking strand C*″* of mCD4-M. (a) Schematic presentation of the mCD4-M peptide topology. Analyzed loops are colored red. (b) Dihedral angles of the loops flanking strand C*″* as function of simulation time are shown in the left and right column, respectively. **φ** angle (C_*i*-1_-N_i_-C*_i_* ^*α*^-C*_i_*) and *ψ* angle (N*_i_*-C*_i_* ^*α*^-C*_i_*-N_*i*+1_) are colored in black and gray, respectively.

 The next analysis focused on the loops flanking strand C*″* in mCD4-M. These loops, which are longer in mCD4 (Figure 1), reside at the gp120 interface and form clashes in the complex of full-length mCD4 (Figure 2). In order to investigate the conformation of these peptide loops upon gp120-binding, the torsion angles of the respective residues were monitored over simulation time. Contrary to the conformationally stable strand C*″*, the flanking loops exhibit much greater fluctuation of their backbone geometry over time (Figure 6). A pronounced flexibility of backbone dihedral angles is detected for residues G41 and K42, located in the C′C*″*-loop, and residues G48 and G49, residing in the C*″*D-loop. The higher loop flexibility also offers an explanation for the absence of clashes in mCD4-M, which are observed for the mCD4 protein (Figure 2). In addition, the conformational flexibility of K42 allows to remove the electrostatic repulsion that is observed between K42 and K429 of gp120 in the complex with the mCD4 protein.

**Figure 7:**
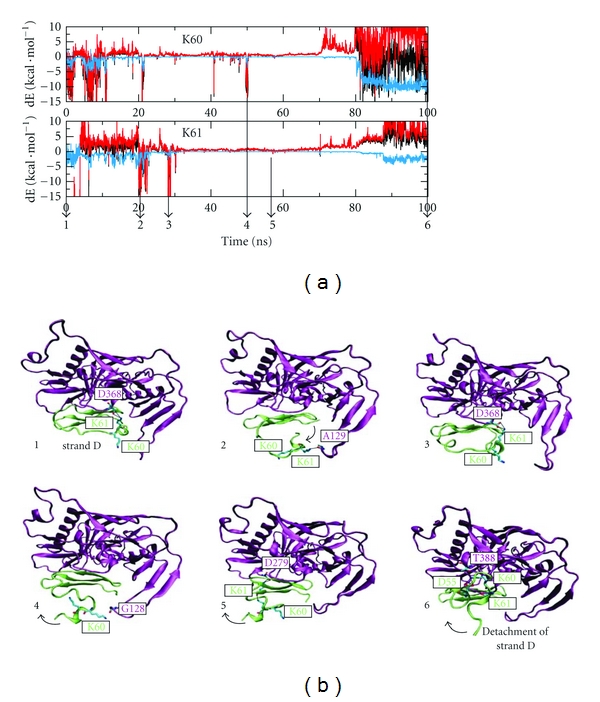
Contacts of K60 and K61 of mCD4-M at the gp120 interface. (a) Interaction energy of K60 and K61 during the simulation. Total energy, electrostatic, and van der Waals contibution are colored in black, blue, and red respectively. (b) Visualization of inter- and intramolecular contacts transiently established by K60 and K61 during simulation. Snapshots were selected at time points when the intermolecular interaction of K60 or K61 was maximal. Peptide and gp120 are colored in green and purple, respectively. Arrows indicate detachment of strand D.

**Figure 8:**
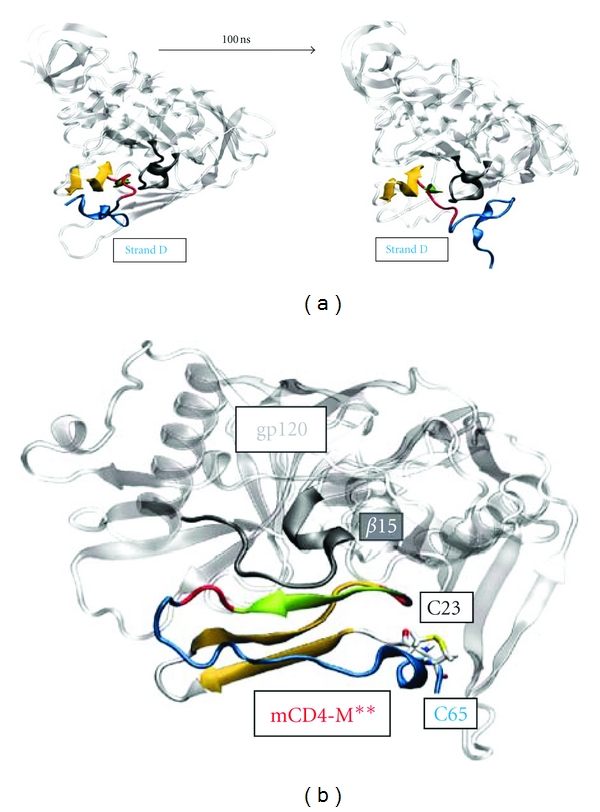
Optimization of mCD4-M. (a) mCD4-peptide-gp120 complex at the beginning (left) and after 100-ns MD simulation (right). For color-coding refer to Figure 4. (b) Visualization of engineered disulfide bond C65–C23 yielding a cyclic peptide with enhanced gp120-binding affinity.

 An enhanced flexibility is also observed for the carboxy-terminal strand D (Figures 7(b) and 8(a)), which is involved in both intramolecular interactions with strand C and in gp120-binding. In contrast to the flanking loops analyzed above, strand D does not only exhibit enhanced local fluctuations but instead becomes completely detached from the mCD4-M core and the gp120 interface. As a consequence, the intermolecular K61-D368 salt-bridge, which is considered an important element of the gp120-CD4 complex, is lost after 6 ns. Consequently, the intrinsic peptide stability is reduced and the gp120-peptide interface weakened. However, transient contacts detected between residues K60/K61 and several alternative gp120 residues may at least partially compensate for the lost K61-D368 salt-bridge (Figure 7).

Although mCD4-M displays a rigid core aligning optimally at the gp120-interface, simulation revealed strand D to detach (Figures 7(b) and 8(a)), thereby destabilizing the interaction. Therefore, we proposed that fixation of strand D to the peptide core might not only enhance intrinsic peptide stability but also contribute to the preservation of interface key contacts such as the K61-D368 salt-bridge. Structural analysis suggested a disulfide bridge that fixes the flexible C-terminus of mCD4-M to the peptide core. Due to their spatial proximity in the initial model, a peptide containing a disulfide bridge between cysteine residues at positions 23 and 65 was expected to enhance peptide stability (Table 2 and Figure 8(b)).

Subsequent binding assays involving this cyclic peptide (mCD4-M**) confirmed that it binds gp120 with a significantly higher affinity than linear mCD4-M (Figure 3), validating the proposed beneficial effect of the conformational constraint introduced by the disulfide bond.

In conclusion, the presented study provides an excellent example of how a combination of computational and experimental methods can be used to shed light on the structural basis of species selectivity in protein-protein interactions, as well as to design and generate molecules with desired qualities. Furthermore, this strategy is of significant potential benefit for the process of structure-based design of synthetic protein mimics, by proposing ways to avoid spatial hindrance, thus improving structural complementarity, and, consequently, affinity between the molecules involved. CD4-derived peptides, which specifically and with high affinity target the CD4-binding site of gp120, are potential candidates for the development of HIV-1 entry inhibitors. The cyclic murine CD4 mimetic peptide proposed here may, therefore, serve as a starting point for the development of such a drug molecule.

## Supplementary Material

The Supplementary Material inclues the following figures and tables:
Figure S1 shows two alternative sequence alignments of murine CD4 with human CD4.
Figure S2 illustrates QMEAN scores and QMEAN Z-scores of the two models mCD4-model 1 and mCD4-model 2.
Figure S3 provides information about model evaluation by ProSA.
Table S1 sums-up all values of model evaluation for the two murine CD4 models (mCD4-model 1 and 2).

## Figures and Tables

**Table 1:** Clashes of mCD4 with gp120. WHAT_CHECK analysis of clashes detected between mCD4 with gp120. All clashes > 0.8 Å are listed; bb and sc denote clashes by backbone or side-chain atoms. The clashes are visualized in Figure 2.

gp120		mCD4		Clash size [Å]
Region	Residue-ID	Residue-ID	Region	
CD4-binding loop	S365 (sc)	G49 (bb)	C*″*D-loop insertion	1.95
*β*20/*β*21 (bridging sheet)	K429 (sc)	K42 (sc)	C′C*″*-loop insertion	1.64
*β*20/*β*21 (bridging sheet)	Q428 (bb)	K42 (sc)	C′C*″*-loop insertion	1.27
CD4-binding loop	I371 (sc)	V44 (sc)	C*″* (F43/V44)	0.87
CD4-binding loop	S365 (sc)	G48 (bb)	C*″*	0.84

**Table 2:** Sequences of CD4 mimetic peptides. hCD4-M and mCD4-M represent the wild-type mimetic peptides presenting residues 22–64 of hCD4 and 22–66 of mCD4, respectively. Residues of hCD4-M and mCD4-M that were replaced by alanine in hCD4-M* and mCD4-M*, respectively, are shown in bold. mCD4-M** represents a cyclic peptide that was proposed based on the computational simulations. The position of the engineered disulfide bond is indicated by brackets next to the involved cysteine residues.

Peptide	Sequence
hCD4-M	Ac-C-Ahx*-^22^KSIQFHWKNSNQIKILGNQGS**F**LTKGPSKLNDRADS**RR**SLWDQ^64^-NH_2_
	Ac-HHHHHH-Ahx-KSIQFHWKNSNQIKILGNQGS**F**LTKGPSKLNDRADS**RR**SLWDQ-NH_2_
hCD4-M*	Ac-C-Ahx-KSIQFHWKNSNQIKILGNQGS**A**LTKGPSKLNDRADS**AA**SLWDQ-NH_2_
mCD4-M	Ac-C-Ahx-^22^KITVFTWKFSDQRKILGQHGKG**V**LIRGGSPSQFDRFDS**KK**GAWEK^66^-NH_2_
mCD4-M*	Ac-C-Ahx-KITVFTWKFSDQRKILGQHGKG**A**LIRGGSPSQFDRFDS**AA**GAWEK-NH_2_

mCD4-M**	Ac-HHHHHH-Ahx-K **[C**TVFTWKFSDQRKILGQHGKG**V**LIRGGSPSQFDRFDS**KK**GAW**C]**K- NH_2_

*Ahx: *ε*-aminohexanoic acid (spacer between tag and peptide sequence).

## References

[1] Dalgleish AG, Beverley PC, Clapham PR (1984). The CD4 (T4) antigen is an essential component of the receptor for the AIDS retrovirus. *Nature*.

[2] Vermeire K, Schols D (2005). Anti-HIV agents targeting the interaction of gp120 with the cellular CD4 receptor. *Expert Opinion on Investigational Drugs*.

[3] Huang CC, Venturi M, Majeed S (2004). Structural basis of tyrosine sulfation and VH-gene usage in antibodies that recognize the HIV type 1 coreceptor-binding site on gp120. *Proceedings of the National Academy of Sciences of the United States of America*.

[4] Kwong PD, Wyatt R, Robinson J, Sweet RW, Sodroski J, Hendrickson WA (1998). Structure of an HIV gp 120 envelope glycoprotein in complex with the CD4 receptor and a neutralizing human antibody. *Nature*.

[5] Kwong PD, Wyatt R, Majeed S (2000). Structures of HIV-1 gp120 envelope glycoproteins from laboratory-adapted and primary isolates. *Structure*.

[6] Pancera M, Majeed S, Ban YE (2010). Structure of HIV-1 gp120 with gp41-interactive region reveals layered envelope architecture and basis of conformational mobility. *Proceedings of the National Academy of Sciences of the United States of America*.

[7] Huang CC, Tang M, Zhang MY (2005). Structural biology: structure of a V3-containing HIV-1 gp120 core. *Science*.

[8] Ryu SE, Kwong PD, Truneh A (1990). Crystal structure of an HIV-binding recombinant fragment of human CD4. *Nature*.

[9] Ryu SE, Truneh A, Sweet RW, Hendrickson WA (1994). Structures of an HIV and MHC binding fragment from human CD4 as refined in two crystal lattices. *Structure*.

[10] Arthos J, Deen KC, Chaikin MA (1989). Identification of the residues in human CD4 critical for the binding of HIV. *Cell*.

[11] Moebius U, Clayton LK, Abraham S, Harrison SC, Reinherz EL (1992). The human immunodeficiency virus gp120 binding site on CD4: delineation by quantitative equilibrium and kinetic binding studies of mutants in conjunction with a high-resolution CD4 atomic structure. *Journal of Experimental Medicine*.

[12] Ashkenazi A, Presta LG, Marsters SA (1990). Mapping the CD4 binding site for human immunodeficiency virus by alanine-scanning mutagenesis. *Proceedings of the National Academy of Sciences of the United States of America*.

[13] Martin L, Stricher F, Misse D (2003). Rational design of a CD4 mimic that inhibits HIV-1 entry and exposes cryptic neutralization epitopes. *Nature Biotechnology*.

[14] Huang CC, Stricher F, Martin L (2005). Scorpion-toxin mimics of CD4 in complex with human immunodeficiency virus gp120: crystal structures, molecular mimicry, and neutralization breadth. *Structure*.

[15] Stricher F, Huang CC, Descours A (2008). Combinatorial optimization of a CD4-mimetic miniprotein and cocrystal structures with HIV-1 gp120 envelope glycoprotein. *Journal of Molecular Biology*.

[16] McClure MO, Sattentau QJ, Beverley PC (1987). HIV infection of primate lymphocytes and conservation of the cd4 receptor. *Nature*.

[17] Landau NR, Warton M, Littman DR (1988). The envelope glycoprotein of the human immunodeficiency virus binds to the immunoglobulin-like domain of CD4. *Nature*.

[18] Maddon PJ, Dalgleish AG, McDougal JS (1986). The T4 gene encodes the AIDS virus receptor and is expressed in the immune system and the brain. *Cell*.

[19] Meier J, Kassler K, Sticht H, Eichler J Exploring species selectivity in protein-protein interactions using synthetic binding site mimetics.

[20] Franke R, Hirsch T, Overwin H, Eichler J (2007). Synthetic mimetics of the CD4 binding site of HIV-1 gp120 for the design of immunogens. *Angewandte Chemie*.

[21] Guex N, Peitsch MC (1997). SWISS-MODEL and the Swiss-PdbViewer: an environment for comparative protein modeling. *Electrophoresis*.

[22] Arnold K, Bordoli L, Kopp J, Schwede T (2006). The SWISS-MODEL workspace: a web-based environment for protein structure homology modelling. *Bioinformatics*.

[23] Peitsch MC (1995). Protein modeling by E-mail. *Nature Biotechnology*.

[24] Kiefer F, Arnold K, Kunzli M, Bordoli L, Schwede T (2009). The SWISS-MODEL repository and associated resources. *Nucleic Acids Research*.

[25] Benkert P, Tosatto SC, Schomburg D (2008). QMEAN: a comprehensive scoring function for model quality assessment. *Proteins*.

[26] Benkert P, Kunzli M, Schwede T (2009). QMEAN server for protein model quality estimation. *Nucleic Acids Research*.

[27] Jaroszewski L, Pawlowski K, Godzik A (1998). Multiple model approach: exploring the limits of comparative modeling. *Journal of Molecular Modeling*.

[28] http://www.jcsg.org/psqs/psqs.cgi/.

[29] Wiederstein M, Sippl MJ (2007). ProSA-web: interactive web service for the recognition of errors in three-dimensional structures of proteins. *Nucleic Acids Research*.

[30] Sippl MJ (1993). Recognition of errors in three-dimensional structures of proteins. *Proteins*.

[31] Hooft RW, Vriend G, Sander C, Abola EE (1996). Errors in protein structures. *Nature*.

[32] Case DA, Darden TA, Cheatham TE (2008). *AMBER10*.

[33] Case DA, Darden TA, Cheatham TE (2010). *AMBER11*.

[34] Hornak V, Abel R, Okur A, Strockbine B, Roitberg A, Simmerling C (2006). Comparison of multiple amber force fields and development of improved protein backbone parameters. *Proteins*.

[35] Jorgensen WL, Chandrasekhar J, Madura JD, Impey RW, Klein ML (1983). Comparison of simple potential functions for simulating liquid water. *The Journal of Chemical Physics*.

[36] Meiselbach H, Sticht H, Enz R (2006). Structural analysis of the protein phosphatase 1 docking motif: Molecular description of binding specificities identifies interacting proteins. *Chemistry and Biology*.

[37] Ryckaert JP, Ciccotti G, Berendsen HJC (1977). Numerical integration of the cartesian equations of motion of a system with constraints: molecular dynamics of *n*-alkanes. *Journal of Computational Physics*.

[38] Humphrey W, Dalke A, Schulten K (1996). VMD: visual molecular dynamics. *Journal of Molecular Graphics*.

[39] Siddiqi MA, Tachibana M, Ohta S (1997). Comparative analysis of the gp120-binding area of murine and human CD4 molecules. *Journal of Acquired Immune Deficiency Syndromes and Human Retrovirology*.

[40] Tachibana M, Siddiqi MA, Ikegami Y (2000). Coreceptor function of mutant human CD4 molecules without affinity to gp120 of human immunodeficiency virus. *Journal of Biological Chemistry*.

[41] Huard B, Mastrangeli R, Prigent P (1997). Characterization of the major histocompatibility complex class II binding site on LAG-3 protein. *Proceedings of the National Academy of Sciences of the United States of America*.

[42] Zhou T, Georgiev I, Wu X (2010). Structural basis for broad and potent neutralization of HIV-1 by antibody VRC01. *Science*.

