# Supplementary material for: Structural Basis for Species Selectivity in the HIV-1 gp120-CD4 Interaction: Restoring Affinity to gp120 in Murine CD4 Mimetic Peptides
Source: Adv Bioinformatics. 2012 Jan 19;2011:736593. doi: 10.1155/2011/736593 (PMC3270550; doi:10.1155/2011/736593)
Supplement: Supplementary file 1 — The Supplementary Material inclues the following figures and tables: Figure S1 shows two alternative sequence alignments of murine CD4 with human CD4. Figure S2 illustrates QMEAN scores and QMEAN Z-scores of the two models mCD4-model 1 and mCD4-model 2. Figure S3 provides information about model evaluation by ProSA. Table S1 sums-up all values of model evaluation for the two murine CD4 models (mCD4-model 1 and 2). [file 736593.f1.pdf]

SUPPLEMENTARY INFORMATION

Structural basis for species selectivity in the HIV-1 gp120 – CD4 interaction: Restoring affinity to gp120 in murine CD4 mimetic peptides

Kristin Kassler[1], Julia Meier[2], Jutta Eichler[2], and Heinrich Sticht[1]

[1]*Bioinformatics, Institute of Biochemistry, Friedrich-Alexander-Universität Erlangen-Nürnberg, Fahrstraße 17, 91054 Erlangen, Germany*

[2]*Medicinal Chemistry, Department of Chemistry and Pharmacy, Friedrich-Alexander-Universität Erlangen-Nürnberg, Schuhstraße 19, 91052 Erlangen, Germany*

Figure S1

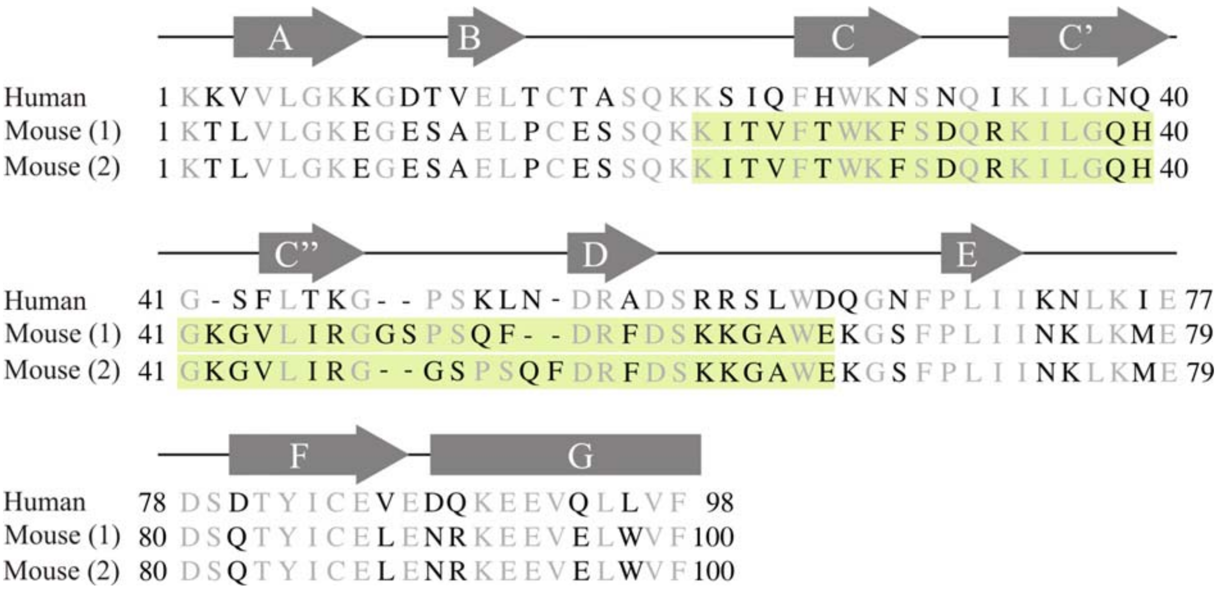

FIGURE S1: Two alternative sequence alignments of murine CD4 with human CD4. The alignments differ in the loop region C''D, which represents part of the gp120 binding site and are based on previous data from the literature: Mouse(1) is aligned according to references [2, 3], while Mouse(2) was aligned according to reference [1]. The stretch covered by mCD4-M (residues 22 to 66) is highlighted in green. Model evaluation (see supplementary data below) revealed that Mouse(1) represents the more suitable alignment for model building. Therefore, this alignment is also shown in Fig. 1a.

TABLE S1: Model evaluation. Evaluation of murine CD4 models (mCD4-model 1, 2) resulting from the different sequence alignments shown in Fig. S1. All structural parameters indicate that model 1 of mCD4 has a superior quality than model 2.

	**mCD4-model 1**	**mCD4-model 2**
QMEAN-score	0.66	0.63
QMEAN Z-score	-1.71	-2.03
PSQS[1]	-0.28	-0.26
ProSA	-6.14	-5.81

[1]typical score for PDB -0.3

Figure S2

a)

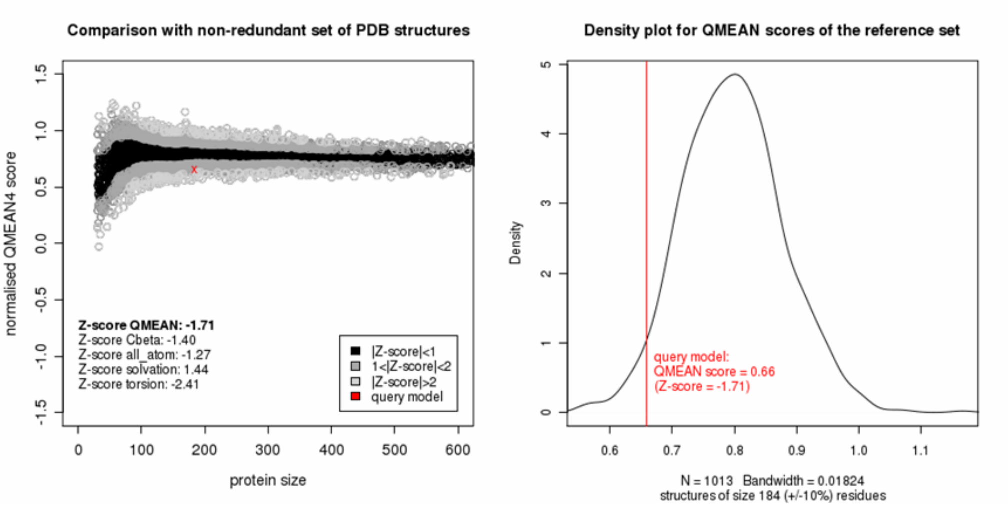

b)

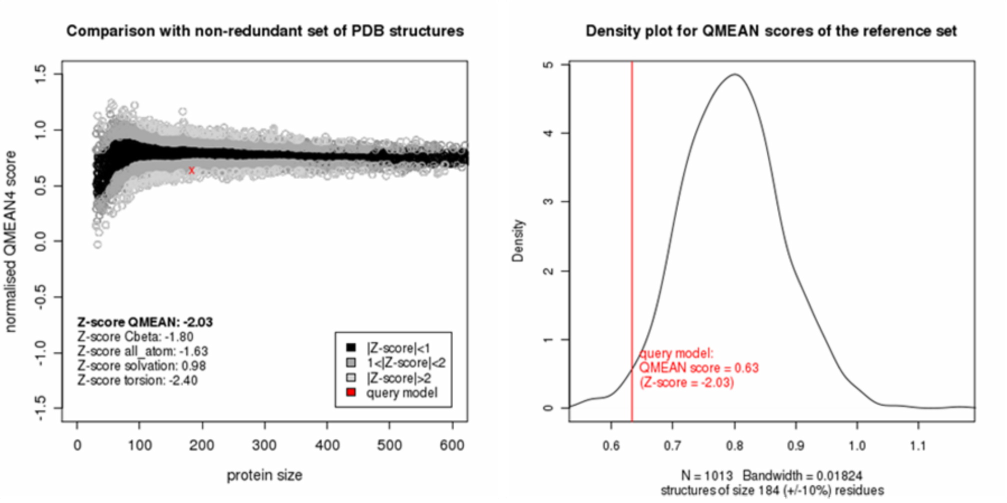

FIGURE S2: QMEAN scores (left) and QMEAN Z-scores (right) of **a**) mCD4-model 1, **b**) mCD4-model 2

Figure S3

a)

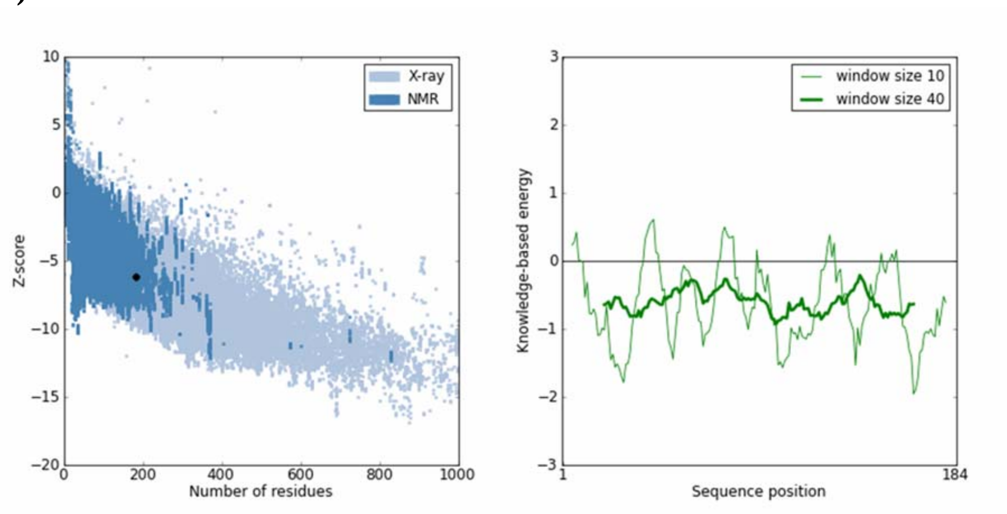

b)

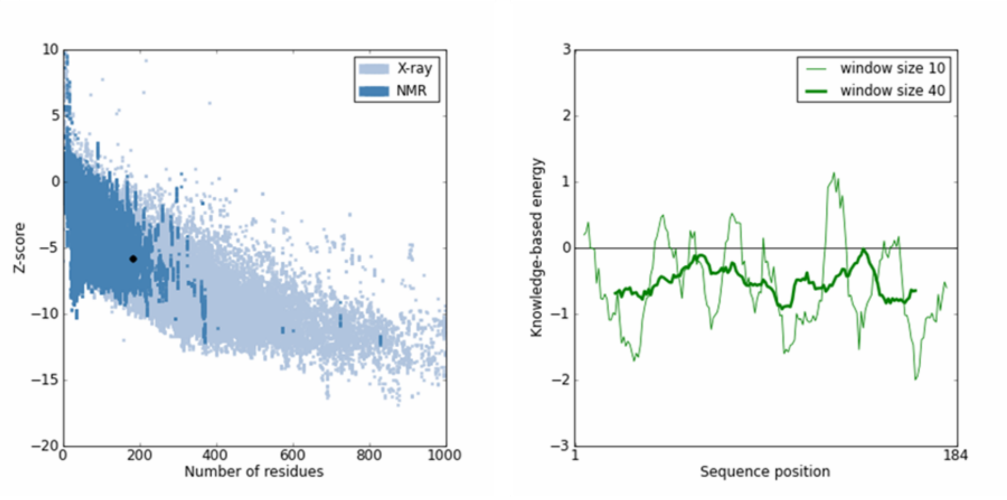

FIGURE S3: ProSA model evaluation of a) mCD4-model 1, **b**) mCD4-model 2. Left: Z-score in comparison to a range of Z-scores characteristic for native protein structures of similar size. Right: per residue energy profile. Low values indicate a favorable energy.

References

1. B. Huard, R. Mastrangeli, P. Prigent, et al., "Characterization of the major histocompatibility complex class II binding site on LAG-3 protein", *Proc Natl Acad Sci U S A*, vol. 94, no. 11, pp. 5744-5749, 1997.
2. M. Tachibana, M. A. Siddiqi, Y. Ikegami, et al., "Coreceptor function of mutant human CD4 molecules without affinity to gp120 of human immunodeficiency virus", *J Biol Chem*, vol. 275, no. 27, pp. 20288-20294, 2000.
3. M. A. Siddiqi, M. Tachibana, S. Ohta, et al., "Comparative analysis of the gp120-binding area of murine and human CD4 molecules", *J Acquir Immune Defic Syndr Hum Retrovirol*, vol. 14, no. 1, pp. 7-12, 1997.
